# STAT3 in Tumor-Associated Myeloid Cells: Multitasking to Disrupt Immunity

**DOI:** 10.3390/ijms19061803

**Published:** 2018-06-19

**Authors:** Yu-Lin Su, Shuvomoy Banerjee, Seok Voon White, Marcin Kortylewski

**Affiliations:** Department of Immuno-Oncology, Beckman Research Institute at City of Hope Comprehensive Cancer Center, Duarte, 91010 CA, USA; yulsu@coh.org (Y.-L.S.); shbanerjee@coh.org (S.B.); sewhite@coh.org (S.V.W.)

**Keywords:** STAT3, myeloid cells, immunosuppression

## Abstract

Myeloid immune cells, such as dendritic cells, monocytes, and macrophages, play a central role in the generation of immune responses and thus are often either disabled or even hijacked by tumors. These new tolerogenic activities of tumor-associated myeloid cells are controlled by an oncogenic transcription factor, signal transducer and activator of transcription 3 (STAT3). STAT3 multitasks to ensure tumors escape immune detection by impairing antigen presentation and reducing production of immunostimulatory molecules while augmenting the release of tolerogenic mediators, thereby reducing innate and adaptive antitumor immunity. Tumor-associated myeloid cells and STAT3 signaling in this compartment are now commonly recognized as an attractive cellular target for improving efficacy of standard therapies and immunotherapies. Hereby, we review the importance and functional complexity of STAT3 signaling in this immune cell compartment as well as potential strategies for cancer therapy.

## 1. Introduction

**Figure 1:**
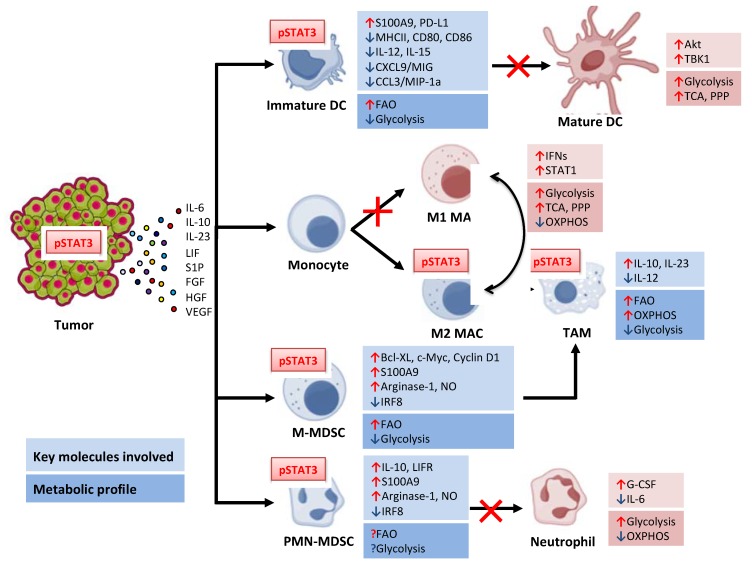
Effects of the tumor microenvironment on myeloid cell differentiation and metabolism. The black arrows indicate the developmental pathway of myeloid cell differentiation. In the presence of tumor-derived factors, the normal developmental pathways to mature DCs, M1 macrophages, or neutrophils are deregulated as indicate by red crosses. These processes result in the accumulation of immature DCs, tumor-associated macrophages, and undifferentiated polymorphonuclear (PMN)- and monocytic(M)-MDSCs. The red and blue arrows indicate up- or down-regulated key molecules and metabolic profiles, the question marks indicate those remain unknown.

In recent years, a number of clinical studies have demonstrated the efficacy of emerging immunotherapies against treatment refractory cancers, thereby gaining the attention of the medical community as well as patients [1,2,3]. In contrast to standard regimens or cytotoxic drugs, immunotherapeutic approaches have an indisputable advantage, being adaptable to a broad range of cancer types. However, emerging clinical results indicate that the responsiveness of patients to new treatments, such as immune checkpoint blockade or chimeric antigen receptor (CAR) T-cells, is still limited by the potentially negative effects of the tumor microenvironment [4,5,6]. Tumor immune evasion is based on complex and intertwined biological, cellular, and molecular mechanisms, which allow cancer cells to escape and also to actively suppress antitumor immunity. A variety of immune cell types present in the tumor microenvironment engage in cross-talk, but most of the time they fail to activate effector cells including T cells, which are necessary for the generation of potent and durable antitumor immune responses (Figure 1). Tumor-associated myeloid cells, such as dendritic cells (DCs), macrophages, and myeloid-derived suppressor cells (MDSCs), play a central role in the cellular immune network based on their physiological role in the regulation of tissue homeostasis, immune surveillance, and wound healing. The tumor microenvironment distorts the function of these myeloid cells, thus promoting chronic inflammation, tumor immune evasion, and neovascularization. As shown in a number of preclinical mouse tumor models and with emerging clinical data, tumor-mediated hijacking of myeloid cell function largely depends on the transcription factor, signal transducer and activator of transcription 3 (STAT3) [7,8]. STAT3 is commonly found to be activated in the majority of human malignancies [9,10,11,12]. The expression of oncogenes like *Src*, *K-ras* and *Bcr-Abl*, growth factors and cytokine receptors can persistently activate STAT3, thereby leading to cellular transformation or promoting cancer cell survival [9]. However, specific cancer cell genetics can alter the outcome of STAT3 signaling, as observed independently in subsets of glioma and prostate tumors. As shown in *Pten* phosphatase-deficient cancer cells, STAT3 can acquire an unexpected role as a tumor suppressor [13,14]. Therefore, an additional level of caution is needed when designing strategies targeting Jak/STAT3 signaling in cancer cells. Nonetheless, targeting the common and functionally well-defined STAT3 in genetically stable, tumor-associated myeloid cells provides for a broadly applicable immunotherapeutic strategy that could overcome the limitations of current cancer immunotherapies [10,15,16].

## 2. Role of STAT3 in Myeloid Cell Differentiation and Activity

One of the hallmarks of the tumor microenvironment is the accumulation of heterogeneous and undifferentiated MDSCs, or partly differentiated but dysfunctional, immature DCs and macrophages [17,18,19]. A lack of adequately mature and fully functional antigen-presenting cells impairs the immune system’s ability to mount an effective anti-tumor response [19]. STAT3 activation, which propagates from cancer cells into non-malignant immune cells infiltrating tumors, is known to play an important role in promoting these tolerogenic effects (Figure 1).

### 2.1. Dendritic Cells

**Figure 2:**
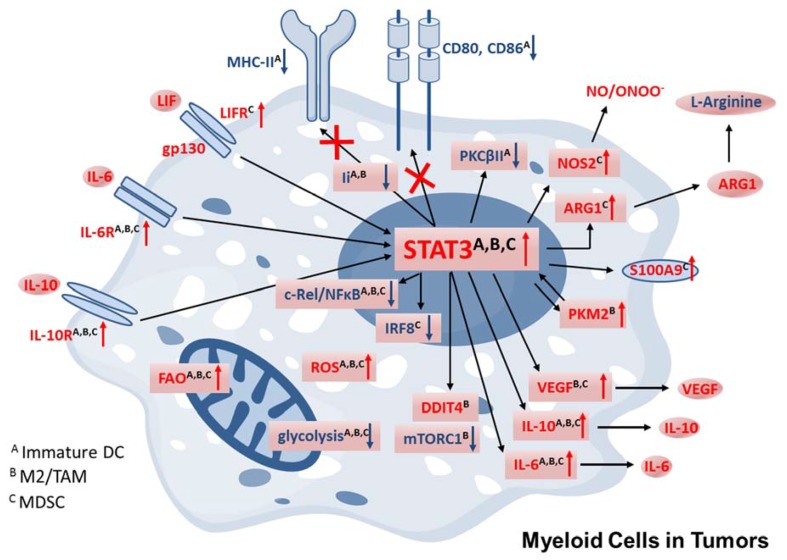
STAT3 orchestrates the immunosuppressive activity of tumor-associated myeloid cells. As indicated by black arrows, persistent activation of STAT3 in myeloid cells in the tumor microenvironment regulates in a positive (red arrows) or negative (blue arrows) manner a number of effector molecules involved in cellular metabolism as well as immunosuppression. The blocked expression of MHC-II, CD80 and CD86 were indicated by red crosses. Some of these mechanisms are common, while others are specific to different myeloid cell subtypes, as indicated in the figure description.

DCs are highly specialized myeloid immune cells that control the activation of adaptive immunity by presenting antigens on major histocompatibility complex (MHC) class I or II molecules to cytotoxic CD8 or helper CD4 T cells, respectively [20]. STAT3 has long been known to be critical in DC generation driven by Fms-related tyrosine kinase (Flt3) ligand, consistent with the lack of DCs in Flt3L-deficient mice [21,22]. Later studies using CD11c-specific *Stat3* deletion found that STAT3 is required primarily for differentiation of plasmacytoid DCs, specialized in type I interferon production, but not the conventional or tissue-resident conventional DCs, at least not at the later stages of their development [23,24]. In contrast, STAT3 activation negatively affects the final steps of DC maturation and critical functions [24,25,26]. Tumors seem to adopt this function of STAT3 by providing an environment rich in activators of this pathway, such as cytokines IL-6, IL-10, growth factors like macrophage colony stimulating factor (M-CSF) or vascular endothelial growth factor (VEGF), or even components of dying cells, including ligands for pattern recognition receptors, e.g., Toll-like receptor 9 (TLR9) that trigger release of IL-6 and/or IL-10 (Figure 1) [27]. While the specific composition of the tumor milieu differs between various cancers, tumor-derived factors commonly induce STAT3 signaling in myeloid cells infiltrating tumors. STAT3 activation results in abnormal accumulation of poorly differentiated myeloid cells, such as MDSCs, discussed later, and immature DCs with a potent tolerogenic effect on T cell immunity. Importantly, STAT3 can inhibit expression of the serine and threonine kinase PKCβII (protein kinase C βII), a kinase crucial for the differentiation of myeloid progenitor cells into DCs (Figure 2) [28]. Tumor-derived factors from human and mouse cancers were shown to induce binding of STAT3 to negative regulatory elements in the promoter of PKCβII gene (*PRKCB*), strongly inhibiting its expression and decreasing expression of DC differentiation markers, such as CD11c, MHC class I and II complexes, and costimulatory molecules. Interestingly, forced expression of PKCβII was able to decrease STAT3 activation by reducing the expression of several STAT3-inducing receptors, such as IL-6Rα, granulocyte colony-stimulating factor (G-CSF) receptor, and VEGF receptor 2 [28]. Thus, conditions increasing PKCβII abundance in myeloid progenitor cells could provide an opportunity to at least partly alleviate tumor-induced immune suppression [28]. Beyond PKCβII, STAT3 is known to target multiple other molecules involved in proinflammatory effects and antigen-presentation. In mouse DCs, IL-6-induced STAT3 was originally shown to upregulate the lysosomal protease, cathepsin S, which then reduced the protein levels of MHC class II molecules as well as invariant chain (Ii) and H2-DM [29]. More recent reports on human macrophages contradicted these early findings. In human macrophages stimulated with IL-10, STAT3 reduced cathepsin S expression, which decreased antigen presentation and T cell activation through impaired Ii processing and thus reduced antigenic peptide loading onto the MHC II dimers [30]. Despite this discrepancy, the negative effect of STAT3 activation on the expression of MHC class II and costimulatory molecules, such as CD80 or CD86, is well established by studies in tumor-associated DCs and other antigen presenting cells (APCs) [31,32,33,34]. STAT3 can also positively regulate the expression of several proteins that suppress DC maturation. These include immunoregulatory molecules, such as the immunoglobulin-like transcript 4 (ILT-4) and specially programmed cell death ligand 1 (PD-L1), which is one of the key tolerogenic molecules on human DCs [35,36]. As shown by studies in hyper-IgE syndrome (HIES) patients, the loss-of-function mutations of STAT3 abrogates the DC responsiveness to IL-10 and thereby impairs their tolerogenic functions and ability to stimulate differentiation of CD4^+^ T cells to regulatory T cells (Tregs) [36]. While the relationship between STAT3 activity in DCs in human cancers and the accumulation of immunosuppressive Tregs has yet to be demonstrated, there is evidence of an inverse correlation between STAT3 inhibition in DCs and reduction of the Tregs in the tumor microenvironment from studies in preclinical mouse tumor models [7,33,37,38]. Mechanistically, such broad and coordinated regulation of DC function can be explained by a differential crosstalk of STAT3 and various types of NF-κB signaling [9,12,39]. STAT3 inhibits NF-κB-mediated proinflammatory effects, especially the expression of IL-12 in DCs as a key costimulatory signal for the induction of Th1 type antitumor immune responses [27,38]. At the same time, it can directly associate with NF-κB complexes, specifically p65/p50 heterodimers, thereby resulting in the upregulation of numerous genes promoting cancer inflammation, supporting cancer cell survival and immune evasion.

### 2.2. Macrophages

Macrophages are key phagocytes in the organism, and show remarkable adaptability to changing conditions of the microenvironment. In response to stimulation by pathogens, products of dying cells, or cytokines, macrophages can differentiate functionally into M1 or M2 phenotypes, as characterized by the release of cytokines promoting Th1 or Th2 responses such as IL-4/IL-10/IL-13 [40]. Endosomal and cytosolic sensors of nucleic acids, such as TLR3/7/8/9, RIG-I, or STING, trigger functional differentiation of macrophages [41,42]. However, these macrophage phenotypes are plastic and allow for the functional adaptation in changing conditions. STAT3 can effectively orchestrate the fine-tuning or even complete reversal of macrophage activity. By synergizing with STAT6, it can promote M2 differentiation, while in parallel STAT3 can antagonize IFN-induced STAT1 activity promoting M1 phenotype [43]. In the tumor microenvironment, dominant and persistent STAT3 activity efficiently suppresses M1 macrophage polarization, dampening cytotoxic and proinflammatory functions including release of IL-12 and induction of Th1 antitumor immune responses [43]. Instead, a higher activation of STAT3 and STAT6 signaling promotes the accumulation of tumor-associated macrophages (TAMs) with strong immunosuppressive and proangiogenic potential [43]. This is at least partly an effect of STAT3 activation shifting the balance from the production of IL-12 with potent proinflammatory and anti-angiogenic effects to IL-23 with immunosuppressive and pro-angiogenic functions [38]. Besides tumor vascularization, STAT3 activity in TAMs seems to promote tumorigenesis and therapeutic resistance by nurturing a population of cancer cells with increased tumorigenic potential, known as cancer stem/initiating cells. In several tumor models including breast, liver, and pancreatic cancers, TAMs were shown to secrete growth factors to induce STAT3-mediated expression of stem cell-related gene expression pattern in cancer cells [44,45,46,47].

### 2.3. Myeloid-Derived Suppressor Cells

Tumors comprise a heterogeneous population of immature MDSCs, which have been associated with poor patient prognosis and tumor progression [48,49,50]. Based on the expression of surface markers and functional differences, human and mouse MDSCs are divided into at least two groups: the polymorphonuclear-MDSCs (PMN-MDSCs) and monocytic-MDSCs (M-MDSCs) [51]. The accumulation of MDSC population results from tumor-driven skewing of monocyte differentiation, which prevents maturation to DCs and macrophages, leading to expansion of M-MDSCs and PMN-MDSCs. The expansion of MDSCs in the tumor microenvironment is induced by cancer-derived cytokines and growth factors, including IL-6, IL-10, VEGF, hepatocyte growth factor (HGF) or G-CSF, which share ability to induce STAT3 signaling [52]. In prostate cancers, another IL-6-type cytokine, leukemia inhibitory factor (LIF), seems to play an important role in the preferential expansion of PMN-MDSCs and their tolerogenic activity [53]. Genetic TCGA analysis indicates that *LIF* is expressed more commonly than *IL6* in human prostate cancers. Importantly, PMN-MDSCs and, to a lesser extent, M-MDSCs isolated from the blood of prostate cancer patients show high surface levels of LIF receptor and respond to LIF stimulation with STAT3 activation and increased T-cell inhibition. Tumor-induced STAT3 plays a central role in regulating both the differentiation and tolerogenic effects of MDSCs. First, STAT3 promotes both expansion and survival of MDSCs through upregulation of Bcl-X_L_, c-Myc, and Cyclin D1 [48]. In addition, MDSC production depends on STAT3-mediated induction of S100A9 calcium-binding proteins on the cell surface. The S100A9 expression interfered with the development of DCs and macrophages, while leading to MDSC accumulation in mice [17,54]. The molecular mechanisms of these effects in human myeloid cells were later shown to depend on the interaction between S100A9 and the immunoreceptor CD33 commonly expressed on myeloid cells, acting as a ligand–receptor pair [55]. Downstream signaling induced by S100A9/CD33 was shown to trigger the expression of crucial immunosuppressive mediators secreted by MDSCs, IL-10, and TGFβ [55]. Finally, STAT3 blocks myeloid cell differentiation by downregulating the expression of IRF8, a transcription factor driving the development of monocytes and DCs while limiting granulocyte development [18,56]. As shown by genetic studies in mice, IRF8 inhibition is in fact responsible not only for differentiation block but also for the expansion of the PMN-MDSC population [18,56]. Similar effects were observed in breast cancer patients, who showed an inverse correlation between IRF8 levels in MDSCs and the MDSC frequency [18]. Conversely, a decrease in STAT3 signaling can enable MDSC differentiation into TAMs, which often become the dominant tumor-infiltrating myeloid cell population [57]. An elegant study by Kumar et al. recently showed that M-MDSCs in the hypoxic tumor express sialin transporter protein to transfer sialic acid to activate the membrane-bound CD45 phosphatase, which in turn dephosphorylates STAT3 [57]. MDSCs utilize multiple strategies to inhibit antitumor immune responses [52]. These include not only the secretion of IL-10 and TGFβ but also complex modulation of metabolic processes regulating reactive oxygen species (ROS) production and amino acid metabolism, as discussed below [58]. Many of the STAT3-dependent mediators generated cytokines and growth factors with a dual role in promoting immunosuppression and stimulating angiogenesis, as described in the case of bFGF, HGF, VEGF, IL-1β, MMP9, CCL2, and CXCL2 [59]. Targeting STAT3 is therefore an attractive strategy to alleviate MDSC-mediated immunosuppression in the tumor microenvironment without the need for myeloid cell depletion.

## 3. STAT3 as a Regulator of Myeloid Cell Metabolism 

Deregulated metabolism in the tumor microenvironment, and more specifically in tumor-associated myeloid cells, has been suggested as an essential element of cancer-related inflammation [60,61]. Rapidly proliferating tumor cells respond to the increased energy consumption by relying on the Warburg effect, which is characterized by strong activation of glycolysis, regardless of the presence of oxygen. As a result of this process, metabolites derived from tumor cells, such as arginine and lactic acid, accumulate in the extracellular space. Such environmental changes can cause reprogramming of immune cell metabolism and redefine cellular functions, including the generation of specific types of immune response [62]. Dramatic changes in cellular metabolism occur during myeloid cell differentiation from monocytes to macrophages, as well as during the development and function of dendritic cells. Emerging evidence points to the critical role of STAT3 in the metabolic regulation of oncogenic and myeloid cell-specific activities.

### 3.1. Dendritic Cells

Dietary conditions, specifically the availability of lipids and their accumulation, can have profound effects on the immunogenic or tolerogenic functions of DCs in cancer [63,64]. In the tumor microenvironment, DCs express elevated surface levels of scavenger receptor-A, which promotes the uptake and accumulation of lipids. As a result, fatty acid oxidation (FAO) becomes a core metabolic process for immature and tolerogenic DCs (Figure 1). The lipid accumulation also negatively affects DC functionality by blocking the loading of antigenic peptides onto MHC class II complexes and impairing antigen presentation and then T cell activation [65]. Oxidized lipids were also shown to inhibit the DC-mediated cross-presentation of peptide antigens on MHC class I molecules [66]. In contrast, DC maturation following stimulation with TLR agonists is associated with a rapid PI3K/Akt-dependent upregulation of aerobic glycolysis [67,68]. Initially, maturating DCs also activate the pentose phosphate pathway (PPP) and tricarboxylic acid (TCA) cycle to produce citrate as a substrate for fatty acid synthesis to ensure building material for growing endoplasmic reticulum and Golgi systems [67]. However, such TLR-induced metabolic transition and DC activation can be effectively abrogated in the presence of IL-10 and likely mediated by STAT3 [25,68,69]. As recently shown in lymphocytes, STAT3 can prevent the expression of the citrate synthase, thereby interrupting a key step in the synthesis of fatty acids and preventing cell growth [70]. Thus, STAT3 plays a gatekeeper role for the metabolic transition of DC in response to immunostimulation. 

### 3.2. Macrophages

During the differentiation of monocytes into tissue macrophages, cells undergo significant epigenetic remodeling of their genome and already at this stage can indicate metabolic differences related to the tolerant or “trained” innate immunity phenotype [71]. Macrophage activation into M1 and M2 subsets results in dramatically different metabolic profiles, with M1 macrophages relying on glycolysis over oxidative phosphorylation (OXPHOS) for energy production and M2 macrophages primarily utilizing FAO and OXPHOS (Figure 1) [61]. While STAT3 has long been suggested to promote M2 macrophage phenotype, a recent report by Ip and colleagues unraveled an elegant molecular mechanism linking the anti-inflammatory effect of IL-10/STAT3 signaling with the direct regulation of cell metabolism [72]. IL-10-induced STAT3 was shown to upregulate DDIT4, a mammalian target of rapamycin (mTOR) inhibitor, thereby inhibiting mTORC1 activation [72]. Since mTOR activity is essential for the transition from OXPHOS to glycolysis, IL-10/STAT3 signaling effectively interrupts this process and limits the inflammatory response. Paradoxically, under chronic inflammatory conditions, persistent STAT3 activation in macrophages can sustain rather than limit their hyperactivated phenotype [73]. In coronary atherosclerosis, the excessive glycolysis and unbalanced ROS production activates ROS-sensitive glycolytic enzyme, pyruvate kinase M2 (PKM2). The PKM2 transfers into the nucleus to directly bind to STAT3, thereby augmenting IL-6 and IL-1β expression and contributing to the pathogenesis of the disease [73]. Thus, the specific outcome of STAT3 activity in macrophages can differ depending on whether it is induced by tightly-regulated cytokine signaling or persistent, chronic inflammatory conditions.

### 3.3. Myeloid-Derived Suppressor Cells

Although normal neutrophils primarily rely on glycolysis, tumor-associated MDSCs, including PMN-MDSCs, were recently shown to depend on FAO for their energy supply [74,75]. Such metabolic reprogramming correlated with the upregulation of lipid uptake receptors, CD36 and MSR1/SR-A, and FAO enzymes, such as carnitine palmitoyltransferase 1 (CPT1) and 3-hydroxyacyl-CoA dehydrogenase (HADHA) [74,75]. Except for CD36, a known STAT3 target gene, it is yet to be established whether STAT3 is directly linked to the metabolic reprogramming occurring in MDSCs [61]. Beyond secreting immunosuppressive cytokines and growth factors, MDSCs make effective use of metabolic processes to deprive T cells of essential metabolites or interfere with their viability and function through the release of ROS [75]. Throughout the evolution of the immune response, arginine metabolism has been key to the catabolic and anabolic process. Myeloid cells are major players that exploit the regulators of arginine metabolism to mediate diverse but often opposing immunological and functional fates. One of the primary immunosuppressive strategies of MDSCs, mainly PMN-MDSCs, is the depletion of arginine, which interferes with T cell activity [48,76]. Arginase-1 (ARG1), the key enzyme in arginine metabolism, is found in macrophages and other myeloid cells, and also in the granular compartment of human granulocytes. In cancer patients, ARG1 was expressed in both circulating and tumor-infiltrating MDSCs. The expression of *ARG1* was shown to be directly regulated by STAT3 binding to multiple sites in the *ARG1* promoter [76]. While inhibition of ARG1 activity partly alleviated immunosuppressive functions of MDSCs, blocking STAT3 using small molecule inhibitors or gene silencing had a more potent effect, connected with a reduction in *ARG1* expression [50,76]. It cannot be ruled out that C/EBPβ, another key transcriptional regulator of myeloid differentiation, can partner with STAT3 to augment *ARG1* expression [48,76]. MDSC in cancer patients and in mice are well known to produce ROS, which can synergize with ARG1 in promoting immunosuppression [77]. The primary mechanism of ROS production in MDSCs is NADPH oxidase (NOS2) activity, with STAT3 regulating the expression of crucial subunits of the NOS2 complex [77]. Generation of ROS (e.g., NO and peroxynitrites, ONOO^−^) results in the nitration of molecules on the surface of T cells when in contact with MDSCs. Importantly, both T cell receptors (TCR) and CD8 molecules can undergo ROS-induced nitration and lose their ability to trigger T cell activation after the formation of the TCR–MHC complex with APCs [78].

## 4. Targeting STAT3 in Myeloid Cells: An Opportunity for Cancer Immunotherapy

STAT3 activation in a variety of tumor-associated myeloid cells generates a powerful and multilayered backing for cancer progression and immune evasion. Therefore, STAT3 provides an exceptional molecular target at the key node of the immune system network, based on the antigen-presenting potential of DCs/macrophages or complex tumor-promoting activity of TAMs and MDSCs. Blocking STAT3 in tumor-associated myeloid cells can permit development of antitumor immune responses, at the same time eliminating tumor resistance to therapeutic assaults. Importantly, inhibition of STAT3 is not cytotoxic to non-malignant cells. The dominant-negative mutations of STAT3 found in patients with autosomal dominant HIES result in the increased susceptibility of patients to infections but are not lethal per se [79,80]. Despite several attempts, no FDA-approved small molecule STAT3 inhibitors exist. Although there are several inhibitors of Janus kinases (JAK) upstream from STAT3, some of the most promising JAK inhibitors showed unexpected neurologic toxicities, such as Wernicke’s encephalopathy, in late clinical studies [81]. These setbacks in the development of JAK inhibitors for cancer therapy may underscore the central role of this kinase family in the regulation of cellular signaling also beyond the immune system. However, even among immune cells there is a need for highly selective approaches to Jak/STAT3 inhibition. The results emerging from a number of preclinical studies indicate that small molecules inhibiting JAK/STAT3 signaling in a broad spectrum of immune cells can impede IFN-mediated antitumor immunity [82] and/or STAT3-mediated generation of memory T cells [83,84]. In addition, the requirement for STAT3 activity is also common for the engineered CAR T cells [85,86]. As recently reported, IL-6/STAT3 gene signature is present specifically in CD19 CAR T-cells from chronic lymphocytic leukemia patients showing complete responses but not in non-responders. In addition, blocking STAT3 abrogated CAR T cell proliferation [86]. These findings emphasize the need for myeloid cell-selective rather than broadly acting STAT3 inhibitors. One of the opportunities for targeting STAT3 specifically in the myeloid cell compartment is delivery using synthetic TLR9 agonists, CpG oligodeoxynucleotide (ODN) [87,88]. Conjugates of CpG ODN with STAT3 inhibitors in the form of siRNA, decoy DNA, or antisense ODN are efficiently internalized by target human and mouse TLR9^+^ myeloid cells in vitro and in vivo. STAT3 inhibition and TLR9 immunostimulation disrupt the tolerogenic effects of the tumor microenvironment and thereby lead to potent antitumor immune responses in a variety of preclinical tumor models in mice [87,89,90,91,92]. Importantly, in cancer patients, TLR9 expression is not limited to plasmacytoid DCs and B cells as in healthy subjects but is upregulated in PMN-MDSCs, which are the dominant tolerogenic cell population accumulating in the blood of patients with various cancers [50,53,93]. As recently demonstrated, CpG-STAT3siRNA conjugates used ex vivo eliminated STAT3-mediated ARG1 expression, thereby alleviating the tolerogenic effect of prostate cancer patients’ PMN-MDSCs on T cell proliferation and activity [50]. With the potential to disrupt the tolerogenic effects of human tumors, CpG-STAT3 inhibitors can be used to stimulate effective presentation of cancer-specific antigens and restore the activity of cytotoxic effector cells. The combination of myeloid cell-specific STAT3 inhibition with other immunostimulatory agents, including TLR3, TLR7, and TLR8 agonists STING or RIG-I, could provide alternative immunotherapeutic strategies and will likely be explored in the future.

## Abbreviations

APC	antigen-presenting cell
ARG1	arginase-1
CAR T-cells	chimeric antigen receptor T-cells
DC	dendritic cell
FAO	fatty acid oxidation
G-CSF	granulocyte colony-stimulating factor
HGF	hepatocyte growth factor
HIES	hyper IgE syndrome
Ii	invariant chain
ILT-4	immunoglobulin-like transcript 4
JAK	Janus kinases
LIF	leukemia inhibitory factor
M-CSF	macrophage colony-stimulating factor
MDSC	myeloid-derived suppressor cell
M-MDSC	monocytic-MDSC
MHC	major histocompatibility complex
mTOR	mammalian target of rapamycin
ODN	oligodeoxynucleotide
OXPHOS	oxidative phosphorylation
PD-L1	programmed cell death ligand 1
PKM2	pyruvate kinase M2
PKCβII	protein kinase C βII
PMN-MDSC	polymorphonuclear MDSC
PPP	pentose phosphate pathway
ROS	reactive oxygen species
STAT3	signal transducer and activator of transcription 3
TAM	tumor-associated macrophage
TCA	tricarboxylic acid
TCR	T cell receptors
Treg	regulatory T cell
TLR9	Toll-like receptor 9
VEGF	vascular endothelial growth factor
